# Identification of Host Gene Transcripts by Machine Learning and Their Application to Predict Outcome in Ebola Virus Disease

**DOI:** 10.1093/infdis/jiag308

**Published:** 2026-06-17

**Authors:** Jocelyn G Pérez, Natasha Y Rickett, Stephan Günther, Romy Kerber, Lisa F P Ng, Tom Fletcher, Tracy MacGill, Todd Myers, Robert Orr, Miles W Carroll, Neil Blake, Julian A Hiscox

**Affiliations:** School of Biodiversity, One Health and Veterinary Medicine, College of Medical, Veterinary and Life Sciences, University of Glasgow, Glasgow, United Kingdom; Medical Research Council-University of Glasgow Centre for Virus Research, Glasgow, United Kingdom; Institute of Infection, Veterinary and Ecological Science, Faculty of Health and Life Science, University of Liverpool, Liverpool, United Kingdom; Institute of Infection, Veterinary and Ecological Science, Faculty of Health and Life Science, University of Liverpool, Liverpool, United Kingdom; Bernhard Nocht Institute for Tropical Medicine, Hamburg, Germany; Bernhard Nocht Institute for Tropical Medicine, Hamburg, Germany; A*STAR Infectious Diseases Labs, Singapore; Liverpool School of Tropical Medicine, Liverpool, United Kingdom; Office of Regulatory and Emerging Science, Food and Drug Administration, Silver Spring, Maryland, USA; Office of Regulatory and Emerging Science, Food and Drug Administration, Silver Spring, Maryland, USA; Office of Regulatory and Emerging Science, Food and Drug Administration, Silver Spring, Maryland, USA; Wellcome Centre for Human Genetics, Nuffield Department of Medicine, Oxford University, Oxford, United Kingdom; Pandemic Sciences Institute, Nuffield Department of Medicine, Oxford University, Oxford, United Kingdom; Institute of Infection, Veterinary and Ecological Science, Faculty of Health and Life Science, University of Liverpool, Liverpool, United Kingdom; Institute of Infection, Veterinary and Ecological Science, Faculty of Health and Life Science, University of Liverpool, Liverpool, United Kingdom; A*STAR Infectious Diseases Labs, Singapore; The Pandemic Institute, Liverpool, United Kingdom

**Keywords:** Ebola virus, EBOV, Ebola virus disease, EVD, medical countermeasure, transcriptome, machine learning

## Abstract

**Background:**

In humans, Ebola virus disease (EVD) has a high mortality rate. While viral load is a known prognostic factor, it is not effective in predicting outcomes across all patients. Exploring host transcriptomic signatures may provide additional predictive value.

**Methods:**

Here, the transcript abundance of 4 candidate host genes (*VCAM1*, *TUBG1*, *MS4A4A*, *ISG15*) was quantified by reverse transcription quantitative polymerase chain reaction (RT-qPCR) in blood samples from hospitalized patients with Ebola virus (EBOV) collected during the 2013–2016 West African outbreak. Samples were taken for diagnostic purposes during the acute phase of infection. Associations between target gene expression, viral load, and outcome (survival/death) were assessed, and machine learning models were trained and tested to predict patient outcome.

**Results:**

EBOV cycle threshold (Ct) values were significantly higher in hospitalized survivors compared to fatalities. During the acute phase, the transcript abundance of *VCAM1* and *MS4A4A* was higher in fatal cases, while *TUBG1* expression was elevated in survivors; *ISG15* expression did not differ by outcome. In support vector machine models, EBOV Ct alone classified patient outcomes with 86.9% accuracy, while *MS4A4A* alone reached 82.6%. Combining EBOV Ct with *MS4A4A* improved prediction to 91.3%. The best-performing model, integrating EBOV Ct, *ISG15*, and *MS4A4A*, achieved 100% accuracy in the test set.

**Discussion:**

Host gene expression markers, particularly *MS4A4A*, in combination with viral load, improved outcome prediction in EVD beyond viral load alone in ambiguous cases with mid-range Ct values. These findings highlight the utility of host transcript-based assays to inform clinical management and for benchmarking the effectiveness of medical countermeasures during outbreaks.

## Teaser

Host markers, particularly *MS4A4A*, add prognostic value to viral load for the clinical management of Ebola virus disease patients and the evaluation of medical countermeasures.

Ebola virus (EBOV) infection in humans causes Ebola virus disease (EVD). Two of the largest recorded filovirus disease outbreaks were in 2013–2016 in West Africa and 2018–2020 in the Democratic Republic of the Congo. Analysis of EBOV outbreaks have identified potential risk factors associated with the severity of EVD, including age, sex, coinfections, and viral load [1, 2]. In the West African outbreak, the abundance of circulating proinflammatory cytokines and chemokines, as well as markers of endothelial dysfunction and coagulopathy, were greater on admission to treatment centers in patients with acute disease who died of EVD compared to those who went on to survive [3]. Likewise, analysis of immunological samples suggested that during the acute phase survivors mounted a robust T-cell response compared to fatal cases [4]. Transcriptomic profiling of blood samples from acutely ill patients with EVD revealed immunological signatures associated with a fatal outcome compared to survival; this included stronger upregulation of interferon signaling and acute-phase responses [5, 6].

There has been a significant drive to determine factors that influence and can be used to predict the clinical outcome of patients with EVD. EBOV load is used as a robust predictor of EVD outcome, although mainly at the lower and higher ranges of cycle threshold (Ct) values. Data from the European Mobile Laboratory (EMLab; deployed to Guinea during the 2013–2016 outbreak) indicated that upon admission to an Ebola treatment center, a low Ct (ie, high viral load) was associated with fatal outcome and a high Ct was associated with survival [1], whereas midrange Ct values were less indicative of patient outcome.

Several statistical models and scoring systems have been developed to help predict EVD outcome (eg, [7–9]). Machine learning approaches have also been applied to sequencing data to identify biomarkers that predict the outcome of patients with EBOV [6]. For example, using blood transcriptomic profiles from patients identified gene sets that correlated with outcome with predictive accuracies of the machine learning models between 85% and 92% [6]. Other machine learning models, combining variables such as viral load, age, body temperature, time to presentation, and clinical symptoms, had predictive accuracies of around 74% [10]. The identification and integration of host biomarkers may help improve prognostic indicators for EVD and aid in the evaluation of medical countermeasures over standard of care. This type of approach was used to evaluate a patient with EVD who was treated with ZMab to determine whether supportive care and application of the medical countermeasure contributed to survival [5].

## METHODS

### RNA Extraction From Control and Clinical Samples

Stored RNA samples extracted from whole blood collected from patients with acute EVD were collected by the EMLab unit deployed in Guinea during the outbreak of EBOV in 2014 and 2015. Virus inactivation, RNA extraction, and rapid diagnostic testing for malaria has been described previously [1]. Venous blood from 5 healthy donors (aged from 29 to 36 years) with no previous history of travel to Africa was collected aseptically in standard EDTA tubes (4 mL) and processed immediately after collection using the QIAamp RNA Blood Mini Kit (Qiagen). For the reverse transcription quantitative polymerase chain reaction (RT-qPCR) assays, RNA samples were first treated with Turbo DNase (Invitrogen). RNA was quantified using Qubit RNA HS Assay Kit (Thermo Fisher Scientific) and read by Qubit Fluorometer (Thermo Fisher Scientific). All samples were tested in duplicate by RT-qPCR.

### Construction of Plasmids

Plasmids containing approximately 1 kb sequence for vascular cell adhesion protein 1 (*VCAM1*), tubulin gamma-1 chain (*TUBG1*), membrane-spanning 4-domains subfamily A member 4A (*MS4A4A*), and <1 kb for interferon-stimulated gene 15 (*ISG15*) inserted into pcDNA3.1(+) downstream of the T7 polymerase promoter were synthesized by GeneArt Gene Synthesis service (Thermo Fisher Scientific).

### Synthesis of RNA Transcripts

Linearization of 5 μg DNA plasmids was performed using the digestion protocol by restriction enzyme NotI-HF (New England Biolabs) and purified using Wizard DNA Clean-Up System (Promega). The in vitro transcription of 1 µg linearized DNA was carried out using MEGAscript T7 Transcription Kit (Thermo Fisher Scientific). DNA template from the in vitro transcripts was removed by digestion with 1 μL TURBO DNase I (Thermo Fisher Scientific) and the purification of transcripts was carried out using the MEGAclear Transcription Clean-Up Kit (Thermo Fisher Scientific). The in vitro transcripts were eluted in 100 µL of RNA storage solution (Thermo Fisher Scientific). The quantity and quality of RNA were assessed using NanoDrop ND-1000 spectrophotometer (Thermo Fisher Scientific). The measurement of RNA copies of the in vitro transcript was determined using a previously developed formula [11]. Ten-fold serial dilutions of RNA transcripts were prepared using RNA storage solution (Thermo Fisher Scientific). To measure the transcript abundance of host genes, a standard curve was created for the RT-qPCR assay. The standard curve consisted of in vitro RNA standards dilutions spanning 10^6^ to 10^1^ copies/µL tested in triplicate by the SYBR Green I-based 1-step RT-qPCR.

### RT-qPCR Assays

For developing the singleplex RT-qPCR assays, primers were designed using RealTimeDesign software (Biosearch Technologies, Novato, USA) to target conserved regions of all available mRNA transcript sequences of each host gene from the National Center for Biotechnology Information’s (NCBI's) reference sequence database (RefSeq) (http://www.ncbi.nlm.nih.gov/RefSeq/) and were synthesized by Eurofin Genomics (Ebersberg, Germany). The sequences of primers, nucleotide position, and amplicon length are presented in Table 1. The optimized conditions for the RT-qPCR were as follows: 5 μL of RNA added to a reaction mixture of 1× iTaq Universal SYBR Green reaction mix (Bio-Rad), 1× iScript reverse transcriptase (Bio-Rad), 100 nM of primer forward, and 100 nM of primer reverse in a final reaction volume of 20 μL. The assay was run on the CFX 96 real-time PCR machine (Bio-Rad). The following thermal cycling parameters were used: 1 cycle of RT for 10 minutes at 50°C followed by 1 cycle of polymerase activation and DNA denaturation at 94°C for 1 minute, and 40 cycles of denaturation at 94°C for 10 seconds, and annealing/extension at 60°C for 30 seconds.

**Table 1.** List of Primer Sets for the 4 Candidate Predictive Gene Transcripts

Gene	NCBI Accession No.	Sequence 5′–3′	Amplicon Length, bp	Nucleotide Position
*VCAM1*	NM_001199834.1	For: CCCGGATTGCTGCTCAGAT	61	997–1057
		Rev: TCACAGCCCATGACACTACA		
*TUBG1*	NM_001070.4	For: GAACGGCTGAATGACAGGTATC	87	861–947
		Rev: GACCACCACATCGCTCATCTC		
*MS4A4A*	NM_148975.2	For: TGAGCCTTAGCATGGGAATAACA	80	362–441
		Rev: CCCGATATACACGGAAATAGGGT		
*ISG15*	NM_005101.3	For: GGCAACGAATTCCAGGTGTC	80	141–220
		Rev: ACGCCGATCTTCTGGGTGATC		

Abbreviations: For, forward; NCBI, National Center for Biotechnology Information; Rev, reverse.

### Statistical Analysis

The nonparametric Mann-Whitney *U* test was used to determine the statistical difference in the transcript abundance of the genes between survivors and fatal cases using GraphPad Prism version 8.3.0. To determine levels of correlation between clinical and molecular variables with disease outcome, continuous variables, including viral load (EBOV), age, and gene expression levels (*VCAM1*, *TUBG1*, *MS4A4A,* and *ISG15*), were tested for normality and analyzed using Pearson correlation coefficient (*r*). The binary outcome variable was coded as 1 (survivor) and 0 (fatal case) to facilitate correlation analysis. To account for missing data, pairwise deletion was applied, and only complete cases were included in each correlation test. Correlation strength was interpreted as follows: weak (|*r*| < 0.3), fair (0.3 ≤ |*r*| < 0.5), moderate (0.6 ≤ |*r*| < 0.8), and very strong (|*r*| ≥ 0.8) [12]. All statistical analyses were conducted using Rstudio version 2024.12.1. The support vector machine (SVM) function in the *e1071* package was used for predicting the outcome of the EVD patients [13]. First, the sample dataset was split in 2 parts: 70% training set used to build the model and 30% set (unseen data) used for evaluating the performance and predictive accuracy of the classifiers. The *caret* package and built-in functions were used to preprocess the training data, train each model, and generate 10-fold cross-validation sets to tune model hyperparameters [14]. The *rose* package was also included during the preprocess of training data to deal with imbalanced classes (unequal number of survivors and fatal cases) [15]. Plots showing the confusion matrix between the EVD outcome predicted by the machine learning models and the actual outcome of the patients were created using the *ggplot2* package.

### Research Ethics

The National Committee of Ethics in Medical Research of Guinea and the Ethics Committee of the Medical Association of Hamburg approved the use of diagnostic leftover samples and corresponding patient data for this study (permits 11/CNERS/14 and PV4910). The collection of samples from healthy donors was approved by the Ethics Committee of the Institute of Infection and Global Health, University of Liverpool (RETH000685).

## RESULTS

### RT-qPCR Assay Development of Prognostic Indicators for Outcome in EVD

To investigate potential prognostic indicators of EVD, the abundance of 4 cellular gene transcripts in blood samples taken from patients with EBOV (during the acute phase) was evaluated to determine whether these correlated with patient outcome. These patients are referred to as hospitalized survivor or hospitalized fatal cases. Three of these transcripts (*VCAM1*, *TUBG1*, *MS4A4A*) were identified previously by machine learning [6]. An additional gene transcript, *ISG15*, was included because it was reported to be upregulated during the acute phase of EVD and correlated with outcome [16, 17]. RT-qPCR assays were developed to identify and measure the abundance of each of the candidate gene transcripts. There was good correlation between Ct value and RNA copy number for all assays (correlation coefficient [*r*^2^] value > 0.99; Supplementary Figure 1). High PCR efficiency was achieved for all genes (> 90.0%), and the slopes of the standard curves were within acceptable range (between −3.3 and −3.6; Supplementary Table 1). The specificity of the assays was evaluated, and no nonspecific products or primer dimers were observed, indicating the specificity of amplification (Supplementary Figure 2).

### Transcript Levels of the Biomarkers Varied According to Patient Outcome

The transcript abundance of *VCAM1*, *TUBG1*, *MS4A4A*, and *ISG15* were determined using the singleplex RT-qPCRs in 81 clinical samples with known disease outcome (36 hospitalized survivors and 45 hospitalized fatal cases; Table 2). All samples were tested in duplicate, with 10 ng of RNA used per PCR.

**Table 2.** Characteristics of the 81 Blood Samples Collected From Patients With EBOV, With Known Clinical Outcome (Survived/Fatal)

ID	EBOV Ct Value	Age, y	Sex	EMLab Site	Year Of Sampling	Comorbidity	Treatment	Outcome
P1	28.45	25	F	Guéckédou	2014	No malaria	No	Survived
P2	29.58	35	F	Guéckédou	2014	No malaria	No	Survived
P3	31.64	40	F	Coyah	2015	No malaria	No	Survived
P4	27.37	54	F	Coyah	2015	No malaria	No	Survived
P5	25.18	15	M	Guéckédou	2014	No malaria	No	Survived
P6	34.57	35	M	Coyah	2015	No malaria	No	Survived
P7	28.10	40	M	Guéckédou	2014	No malaria	No	Survived
P8	27.65	51	M	Guéckédou	2014	No malaria	No	Survived
P9	27.95	5	F	Coyah	2015	No malaria	No	Survived
P10	16.12	10	F	Guéckédou	2014	No malaria	No	Fatal
P11	17.07	25	F	Coyah	2015	No malaria	No	Fatal
P12	18.25	30	F	Guéckédou	2014	No malaria	No	Fatal
P13	17.08	40	F	Coyah	2015	No malaria	No	Fatal
P14	16.34	50	F	Guéckédou	2014	No malaria	No	Fatal
P15	20.98	55	F	Guéckédou	2014	No malaria	No	Fatal
P16	16.59	65	F	Guéckédou	2014	No malaria	No	Fatal
P17	15.59	9	M	Guéckédou	2014	No malaria	No	Fatal
P18	17.91	20	M	Guéckédou	2014	No malaria	No	Fatal
P19	15.12	30	M	Guéckédou	2014	No malaria	No	Fatal
P20	16.72	40	M	Guéckédou	2014	No malaria	No	Fatal
P21	16.22	50	M	Guéckédou	2014	No malaria	No	Fatal
P22	18.99	55	M	Guéckédou	2014	No malaria	No	Fatal
P23	15.99	65	M	Guéckédou	2014	No malaria	No	Fatal
P24	17.16	70	M	Guéckédou	2014	No malaria	No	Fatal
P25	19.10	58	F	Guéckédou	2014	No malaria	No	Survived
P26	17.25	55	M	Guéckédou	2014	No malaria	No	Survived
P27	17.09	40	F	Guéckédou	2014	No malaria	No	Survived
P28	19.08	18	M	Guéckédou	2014	No malaria	No	Survived
P29	17.73	24	M	Guéckédou	2014	No malaria	No	Survived
P30	19.76	70	F	Guéckédou	2014	No malaria	No	Survived
P31	19.04	21	M	Guéckédou	2014	No malaria	No	Survived
P32	14.42	12	M	Guéckédou	2014	No malaria	No	Survived
P33	23.19	70	M	Guéckédou	2014	No malaria	No	Survived
P34	19.54	5	F	Guéckédou	2014	No malaria	No	Survived
P35	16.08	25	F	Guéckédou	2014	No malaria	No	Survived
P36	21.29	60	M	Guéckédou	2014	No malaria	No	Survived
P37	15.99	11	F	Guéckédou	2014	No malaria	No	Survived
P38	19.35	30	F	Guéckédou	2014	No malaria	No	Survived
P39	17.71	10	M	Guéckédou	2014	No malaria	No	Survived
P40	18.50	30	M	Guéckédou	2014	No malaria	No	Survived
P41	16.52	3	F	Guéckédou	2014	No malaria	No	Survived
P42	17.44	19	F	Guéckédou	2014	No malaria	No	Survived
P43	15.24	60	F	Guéckédou	2014	No malaria	No	Survived
P44	17.24	6	M	Coyah	2015	No malaria	No	Survived
P45	19.55	16	F	Guéckédou	2014	No malaria	No	Survived
P46	19.95	45	F	Guéckédou	2014	No malaria	No	Survived
P47	19.31	3	M	Guéckédou	2014	No malaria	No	Survived
P48	19.81	45	M	Guéckédou	2014	No malaria	No	Survived
P49	14.66	75	F	Guéckédou	2014	No malaria	No	Fatal
P50	14.61	45	F	Guéckédou	2014	No malaria	No	Fatal
P51	15.30	48	M	Guéckédou	2014	No malaria	No	Fatal
P52	15.51	18	M	Guéckédou	2014	No malaria	No	Fatal
P53^a^	13.50	14	F	Guéckédou	2014	Malaria^a^	No	Fatal
P54	14.91	38	F	Guéckédou	2014	No malaria	No	Fatal
P55	19.95	30	F	Guéckédou	2014	No malaria	No	Fatal
P56	16.69	0.6	M	Guéckédou	2014	No malaria	No	Fatal
P57	14.99	6	F	Guéckédou	2014	No malaria	No	Fatal
P58	14.60	24	M	Guéckédou	2014	No malaria	No	Fatal
P59	15.34	30	M	Guéckédou	2014	No malaria	No	Fatal
P60	15.07	50	F	Guéckédou	2014	No malaria	No	Fatal
P61	14.03	15	M	Guéckédou	2014	No malaria	No	Fatal
P62	15.33	6	M	Guéckédou	2014	No malaria	No	Fatal
P63	15.82	38	M	Guéckédou	2014	No malaria	No	Fatal
P64	12.87	53	M	Guéckédou	2014	No malaria	No	Fatal
P65	16.33	60	F	Guéckédou	2014	No malaria	No	Fatal
P66	15.40	45	M	Guéckédou	2014	No malaria	No	Fatal
P67	17.44	20	F	Guéckédou	2014	No malaria	No	Fatal
P68	19.91	80	F	Coyah	2015	No malaria	No	Fatal
P69	16.26	59	M	Coyah	2015	No malaria	No	Fatal
P70	15.20	25	F	Guéckédou	2014	No malaria	No	Fatal
P71	16.22	60	M	Coyah	2015	No malaria	No	Fatal
P72	19.10	24	F	Guéckédou	2014	No malaria	Favipiravir	Fatal
P73	23.00	40	F	Guéckédou	2014	No malaria	Favipiravir	Fatal
P74	23.64	60	F	Guéckédou	2014	No malaria	Favipiravir	Fatal
P75	17.41	29	M	Guéckédou	2014	No malaria	Favipiravir	Fatal
P76	17.53	38	M	Guéckédou	2014	No malaria	Favipiravir	Fatal
P77	26.42	14	F	Guéckédou	2014	No malaria	Favipiravir	Fatal
P78	20.42	18	F	Guéckédou	2014	No malaria	Favipiravir	Fatal
P79	22.17	50	F	Guéckédou	2014	No malaria	Favipiravir	Survived
P80	27.15	18	M	Guéckédou	2014	No malaria	Favipiravir	Survived
P81	29.15	62	M	Guéckédou	2014	No malaria	Favipiravir	Survived

The treatment column indicates those patients who received standard of care (No) or those treated with favipiravir.

Abbreviations: Ct, cycle threshold value; EBOV, Ebola virus; EMLab, European Mobile Laboratory; F, female; M, male.

^a^Positive malaria test by rapid diagnostic test.

Transcript abundance density of all genes quantified by RT-qPCR in all samples was determined (Figure 1). There was a clear separation in transcript abundance between samples collected from healthy donors (control) and from patients with EBOV. However, there was some separation but also overlap between the survivor and fatal cohorts in the expression levels of *ISG15*, *VCAM1*, *TUBG1*, and *MS4A4A*.

**Figure 1.**
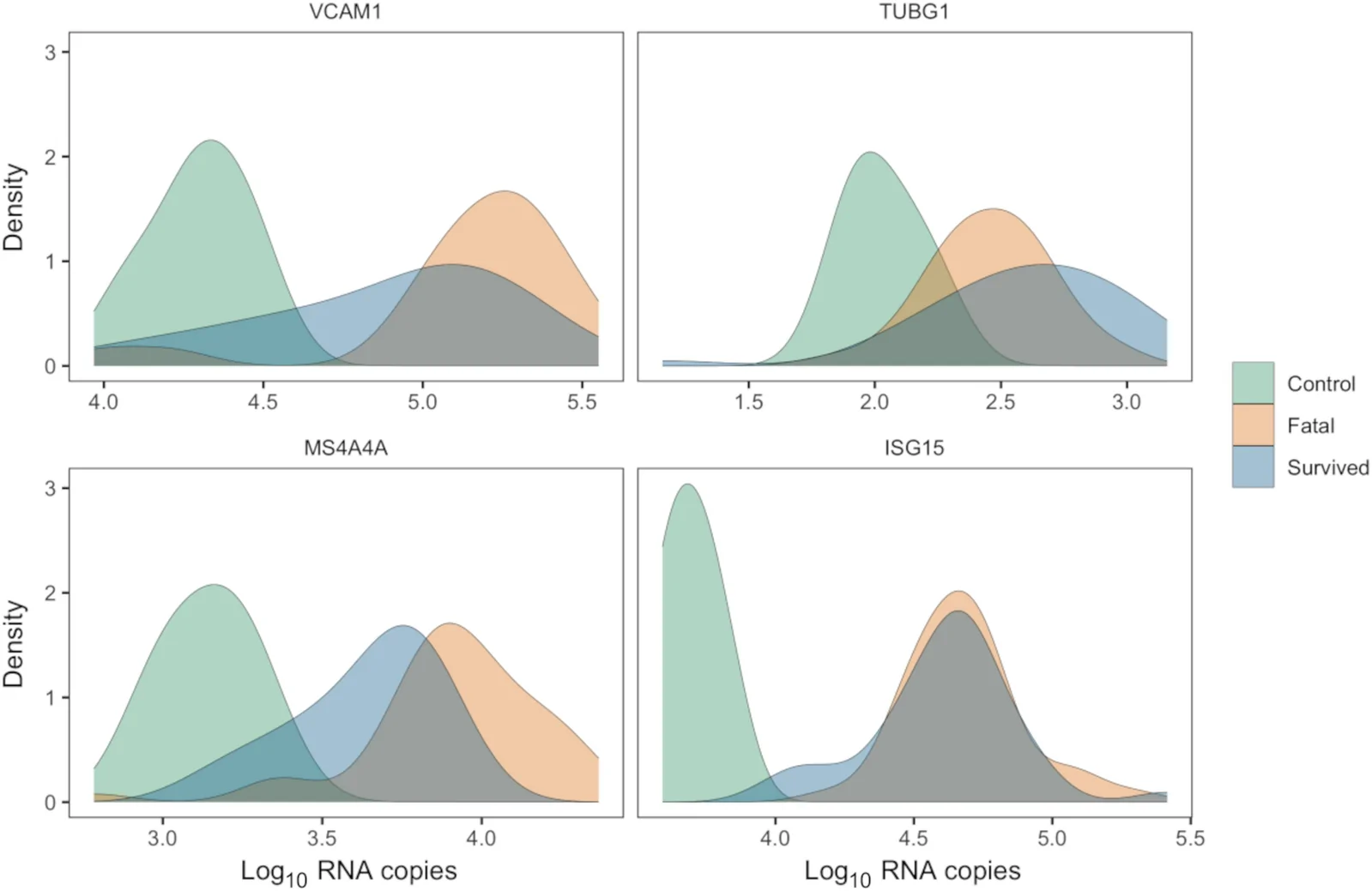
Density plots of the transcript abundance of the candidate predictive genes tested by reverse transcription quantitative polymerase chain reaction (RT-qPCR), taken from 81 blood samples of patients with Ebola virus (36 hospitalized survivors and 45 hospitalized fatal cases) and 5 samples taken from healthy donors.

Data collected from individuals with EBOV in this study showed a significant difference (*P* < .0001) in the EBOV Ct values reported from hospitalized survivors and hospitalized fatal cases during the acute phase (Figure 2*A*). Hospitalized fatal cases had an average Ct value of 17 (median 16, range 13–26) compared to a mean Ct of 22 (median 20, range 14–35) in hospitalized survivors. Hospitalized fatal cases had a 2-fold increase in the abundance of *VCAM1* and *MS4A4A* transcript copies compared to hospitalized survivors (Figures 2*B* and 2*D*; *P* < .0001). In contrast, the transcript abundance of *TUBG1* was 2 times higher in hospitalized survivors compared to hospitalized fatal cases (Figure 2*C*; *P* = .0181). In this cohort of patients, there was no significant difference in the transcript abundance of *ISG15* between hospitalized survivors and hospitalized fatal cases (Figure 2*E*; *P* = .2824). These trends can be clearly seen when EBOV Ct is plotted against gene transcript abundance (Supplementary Figure 3).

**Figure 2.**
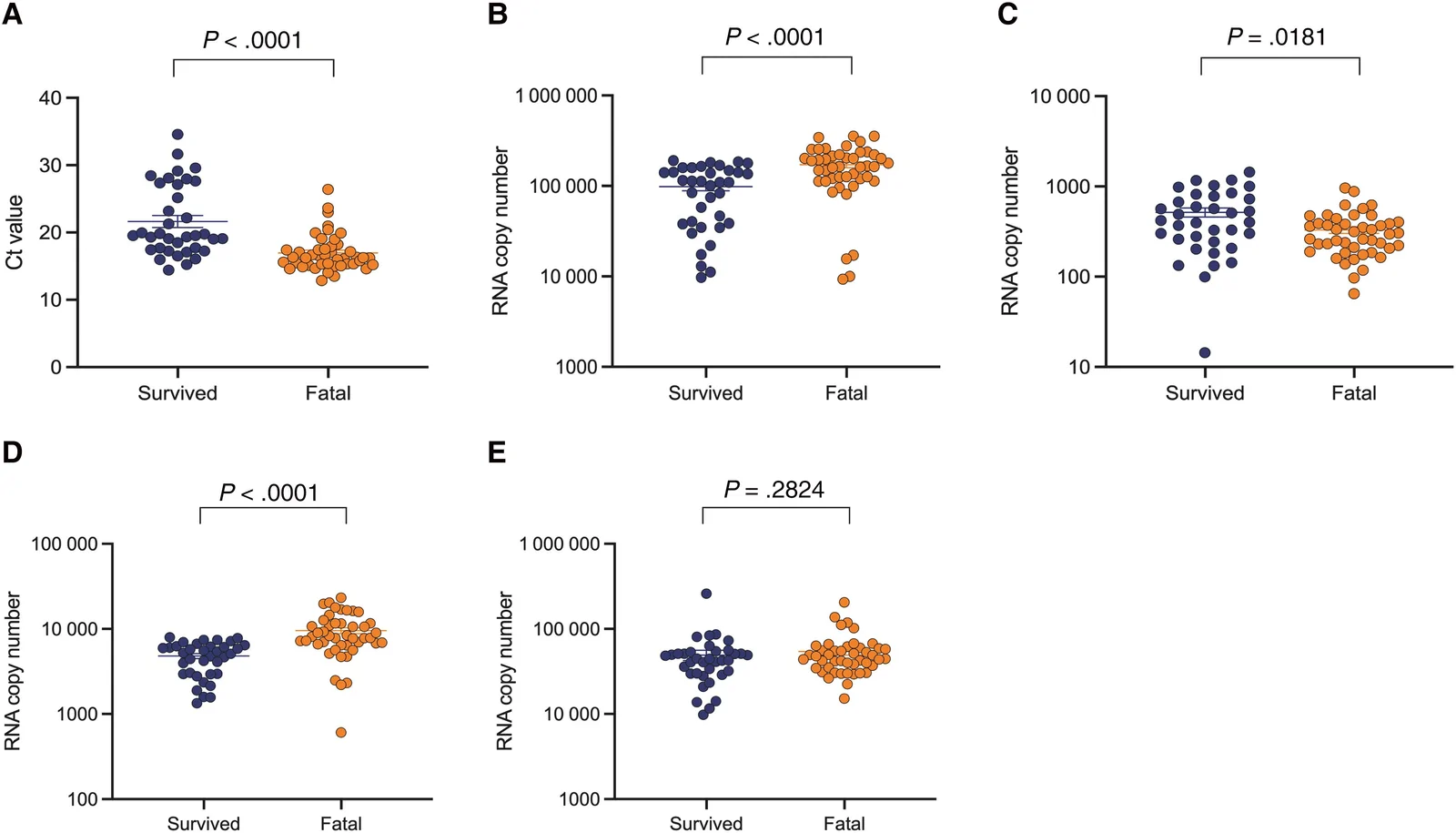
Comparison of viral load and transcript abundance in hospitalized survivor or fatal cases. EBOV cycle threshold (Ct) values (*A*) and transcript abundance of *VCAM1* (*B*), *TUBG1* (*C*), *MS4A4A* (*D*), and *ISG15* (*E*) are compared. Each dot represents an individual (survivor [n = 36], fatal case [n = 45]). The bars indicate the mean and SEM. Statistical differences between hospitalized survivors and hospitalized fatal cases were evaluated using the nonparametric Mann-Whitney *U* test.

A correlation analysis was performed on the 81 samples from patients with EBOV. A higher EBOV Ct value, that is lower viral load, correlated with the hospitalized survivor cases (*P* < .0001), whilst any correlation with age and *ISG15* expression was weak and not statistically significant (*P* = 1.00 for both; Supplementary Table 2). A greater expression of *VCAM1* and *MS4A4A* transcripts correlated with hospitalized fatal cases (*P* < .001 and *P* < .0001, respectively). Conversely, higher *TUBG1* expression correlated with hospitalized survivors (*P* = .02). Bonferroni adjusted *P* values were reported to correct for multiple comparisons.

During the 2013–2016 outbreak in West Africa, a number of patients were treated with favipiravir, with a modest, though not statistically significant, beneficial effect reported [18]. To explore the potential role of favipiravir treatment in patient outcome, a correlation analysis was performed on the dataset. The correlation between patient outcome and EBOV load, *VCAM1,* and *MS4A4A* expression was classified as moderate (Supplementary Table 3). Due to the limited sample size of patients who received favipiravir, exploring this relationship through logistic regression was not appropriate. However, it was clear that transcript abundance across the test genes was comparable between those who received favipiravir and those who did not.

### Evaluation of Different Machine Learning Algorithms to Predict Patient Outcome

The Boruta algorithm (which allows a ranking of features) was used to down-select appropriate variables for inclusion in the predictive models, to improve their accuracy (Figure 3). The attributes that were relevant for the prediction of patient outcome were *MS4A4A*, EBOV Ct, *VCAM1*, *TUBG1*, and *ISG15*. Interestingly, *MS4A4A* transcript abundance had higher Z-score values than EBOV Ct, suggesting that it may have better performance as a predictor variable than the proxy for EBOV load, at least in this dataset. Age was found to be a non-relevant attribute in this dataset and therefore was not taken forward in the model building.

**Figure 3.**
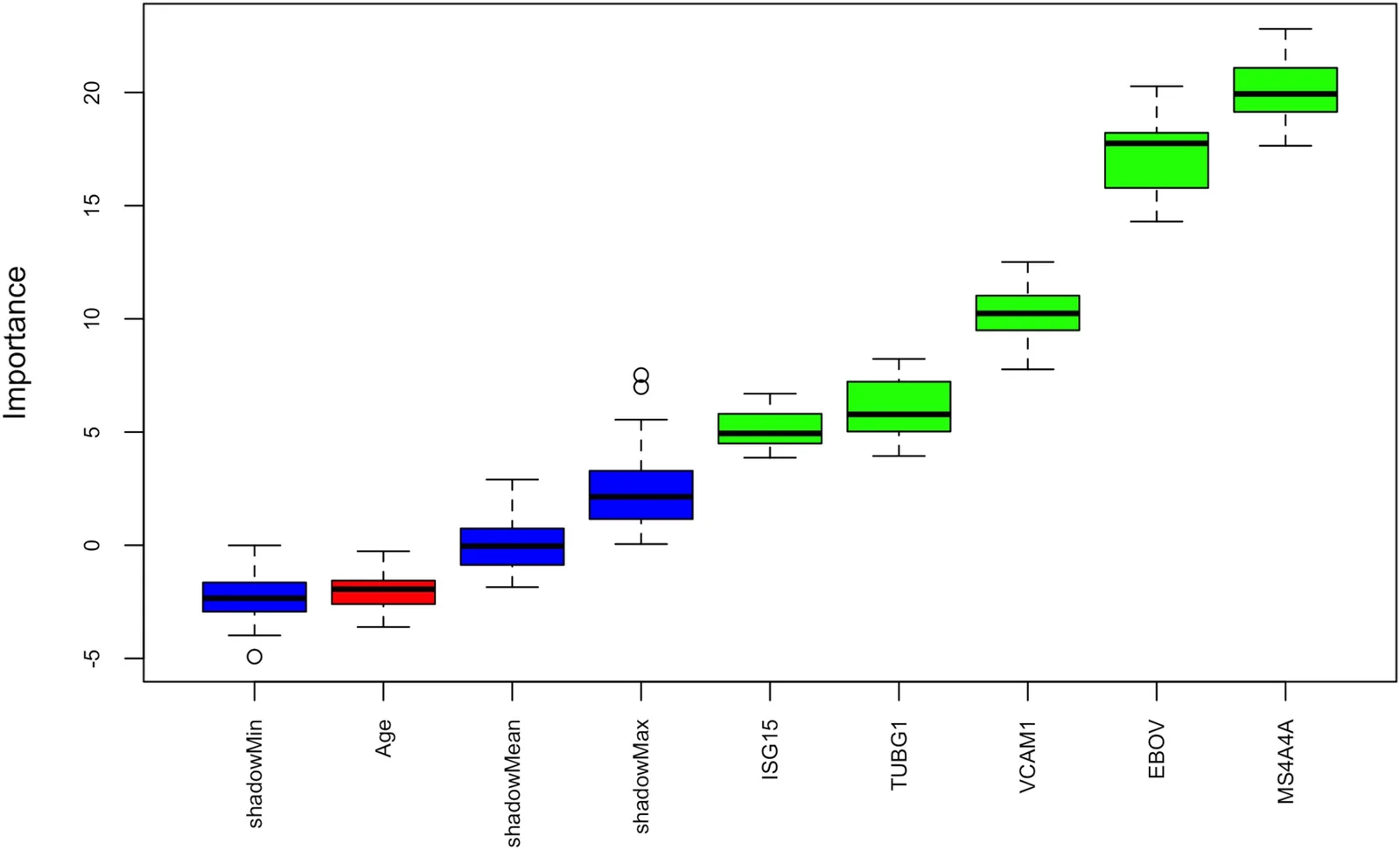
Selection of key predictor variables for inclusion in models predicting patient outcome in those with Ebola virus using a Boruta result. The y-axis shows importance in terms of Z-scores. Blue boxplots correspond to minimum, average and maximum Z score of a shadow attribute. Green boxplots represent Z scores of the confirmed important attributes. Red boxplot represents a rejected attribute. Box plot features are as follows: circles are outliers, dashed-lines whiskers are 1.5 times the interquartile range, the rectangle represents the first and third quartiles, and the horizontal bar is the median.

Different algorithms (linear discriminant analysis, logistic regression, naive Bayes, k-nearest neighbors, support vector machine [SVM], classification and regression tree [CART], bagged CART, random forest, C5.0, and generalized boosted modelling) were spot checked to identify the most appropriate machine learning techniques to employ for building this model. The full dataset was split into 70% for training (58 individuals consisting of 26 hospitalized survivors and 32 hospitalized fatal cases) and 30% for testing (23 individuals consisting of 10 hospitalized survivors and 13 hospitalized fatal cases) with repeated cross-validation with 10-fold and 3 repeat functions. Three models were the most accurate classifiers: SVM (86% accuracy, κ = 0.71), k-nearest neighbors (85% accuracy, κ = 0.71), and random forest (84% accuracy, κ = 0.66) (Supplementary Figure 4). The SVM model was selected for further refinement.

### Optimization of SVM Increased Accuracy of Predictive Model

To evaluate the final performance of the model, the prediction was performed on the testing set (23 individuals consisting of 10 hospitalized survivors and 13 hospitalized fatal cases). An SVM model that only used EBOV Ct value as the predictor variable correctly classified 86.9% (95% CI, .6641–.9722) of patients from the testing set (Figure 4*A*). Only 1 hospitalized fatal case was misclassified with this model; 12 of 13 (92.3%) hospitalized fatal cases and 8 of 10 (80.0%) hospitalized survivors were correctly classified. Among the models built with a single gene transcript, the *MS4A4A* model had the best performance and correctly classified 82.6% (95% CI, .6122–.9505) of patients from the testing set (Figure 4*B*). This model correctly classified 12 of 13 (92.3%) hospitalized fatal cases and 7 of 10 (70.0%) hospitalized survivors. *VCAM1*, *ISG15*, and *TUBG1* models had lower predictive accuracy (69.5%, 65.2%, and 73.9%, respectively; Supplementary Figure 5*A*, 5*B*, and 5*C*). The combination of EBOV Ct and *MS4A4A* in a model improved the performance to 91.3% accuracy (95% CI, .7196–.9893) and correctly classified all hospitalized fatal cases and 8 of 10 (80.0%) of hospitalized survivors (Figure 4*C*). Another model combining *VCAM1*, *MS4A4A*, and *ISG15* had a high predictive accuracy of 91.3% (95% CI, .7196–.9893). This model misclassified only 1 hospitalized fatal case and 1 hospitalized survivor (Supplementary Figure 6).

**Figure 4.**
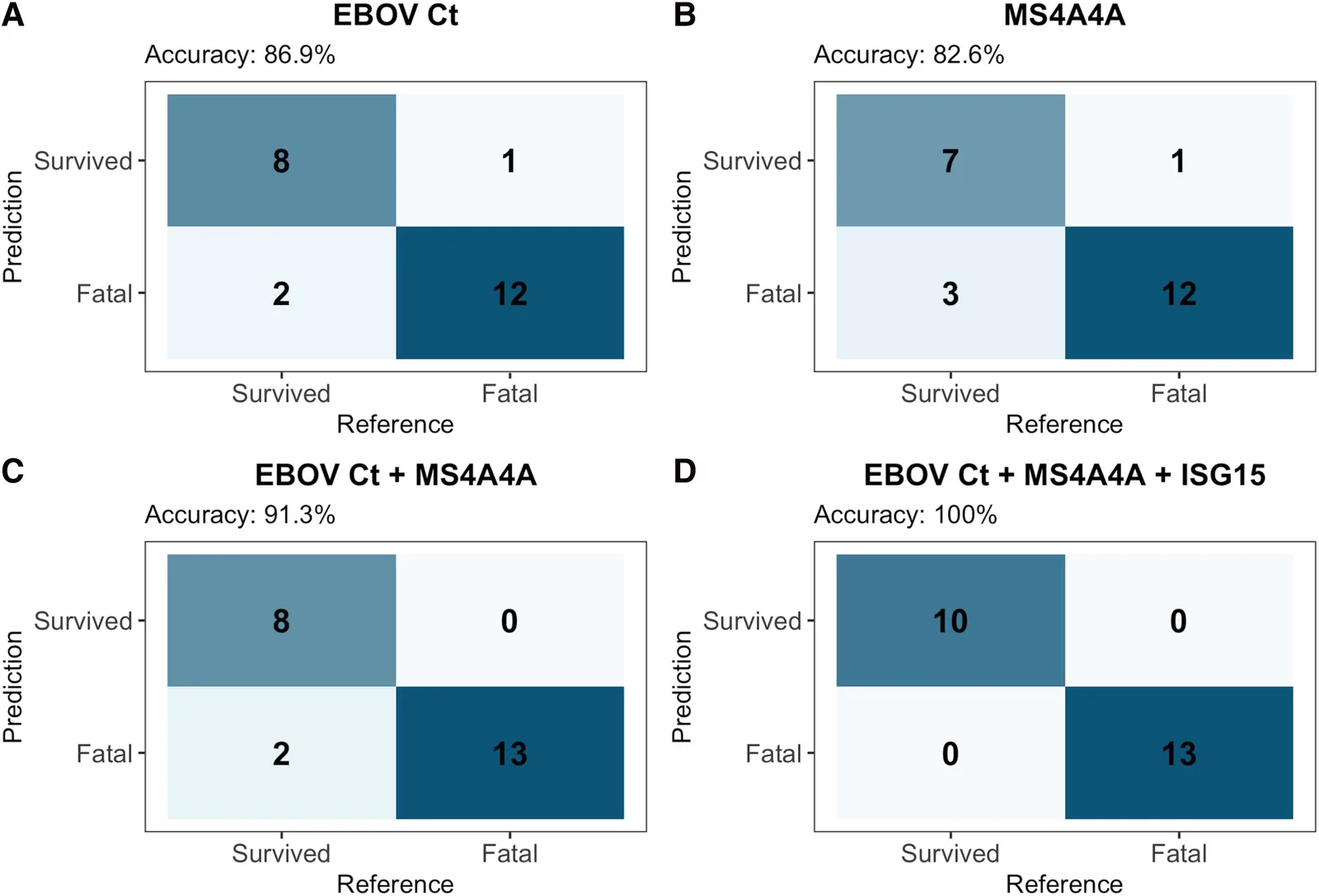
*A*–*D*, Confusion matrix showing support vector machine models with the best predictive performance on the testing set and built with few predictor variables. The confusion matrix reports the predicted and actual outcome of the testing set (23 patients). The intensity of the background color corresponds to the frequency of patients. The accuracy metric is defined as the fraction of patients that are correctly classified. Abbreviations: Ct, cycle threshold value; EBOV, Ebola virus.

Between all the possible combinations of predictor variables, the model combining EBOV, *ISG15*, and *MS4A4A* variables had the best performance and was 100% (95% CI, .8518–1) accurate classifying the hospitalized survivors and the hospitalized fatal cases from the testing set (Figure 4*D*). Replacing *MS4A4A* with *VCAM1* in the model also showed a good performance of 95.6% (95% CI, .7805–.9989). This model classified all hospitalized survivors with only 1 hospitalized fatal case misclassified. The same accuracy was obtained for a model combining EBOV, *MS4A4A*, and *VCAM1*, which classified all hospitalized fatal cases and misclassified only 1 hospitalized survivor. Using all 4 proposed predictor variables (EBOV Ct, *VCAM1*, *ISG15*, *MS4A4A*, and *TUBG1*) in a model classified all hospitalized survivors correctly, with only 1 hospitalized fatal case misclassified (Supplementary Figure 7*A*, 7*B,* and 7*C*).

### Host Markers Outperformed or Added Prognostic Value to Viral Load in Ambiguous Cases With Mid-range Ct Values

To corroborate whether the performance of the models reflected the incremental prognostic value of the host markers independent of viral load, a subset of patients from the same cohort with intermediate Ct values were additionally tested, which are considered to be within the clinical grey zone, that is where viral load does not correlate with outcome. The new testing set was stratified in a range of Ct 19–21 (n = 15) corresponding to approximately 60%–40% case fatality ratio from the outbreak in Guinea [1]. The model built with *MS4A4A* as a standalone predictor had an accuracy of 73.3% (95% CI, .449–.9221) and outperformed EBOV Ct model with 66.6% accuracy (95% CI, .3838–.8818). This failed to classify correctly all fatal cases from the testing set (Figure 5*A* and 5*B*). A lower predictive accuracy was observed for the models built with *VCAM1* (66.6%; 95% CI, .3838–.8818), *TUBG1* (53.3%; 95% CI, .2659–.7873), and *ISG15* (40%; 95% CI, .1634–.6771; Supplementary Figure 8*A*, 8*B,* and 8*C*). The predictive performance increased in a model built with *MS4A4A* and EBOV Ct, reaching 93.3% accuracy (95% CI, .6805–.9983) and 100% when all host markers were combined with viral load (95% CI, .782–1) (Figure 5*C* and 5*D*).

**Figure 5.**
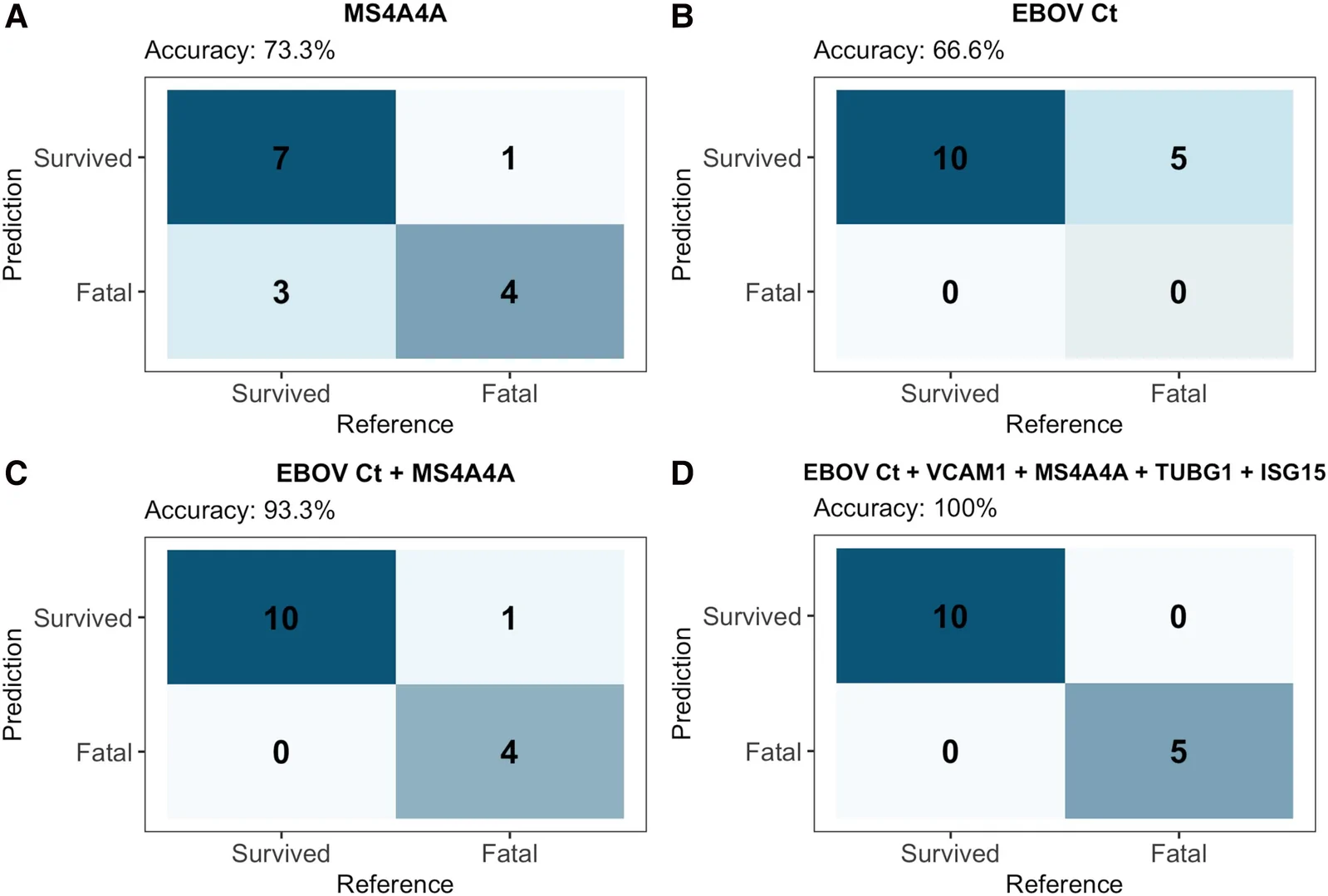
*A*–*D*, Confusion matrix showing predictive performance of the models on the new testing set. The confusion matrix reports the predicted and actual outcome of the testing set (15 patients) that had mid-range Ct values. Abbreviations: Ct, cycle threshold value; EBOV, Ebola virus.

## DISCUSSION

In resource-limited settings, supportive care is often the only option for those with EBOV, which has improved during and following the 2013–2016 West African outbreak [7, 19, 20]. Application of medical countermeasures in resource-limited settings can be challenging, depending on the scale of the outbreak, and are given alongside standard of care. Generally, the associated humanitarian considerations can rule out the opportunity for randomized comparisons [21]. Observational data are used to evaluate the efficacy of these treatments [21]. Therefore, formally demonstrating the efficacy of medical countermeasures would be useful to aid licensure decisions.

We have previously used high-resolution sequencing of diagnostic leftover blood samples to characterize the host response in West African patients with EVD [6]. The diagnostic sample was taken when the patient was acutely ill (ie, ill enough to present to a treatment center). The mean time to the onset of symptoms for the hospitalized survivors was 6.4 days and for hospitalized fatal cases was 5.9 days, with a range of 2–19 and 2–22 days, respectively [6]. Viral load information gathered at this time point can also be used to provide an indication of outcome. This is accurate at high Ct values (indicative of survival) or low Ct values (indicative of a fatal outcome) [1]. Whilst Ct is a good indicator of outcome it is not infallible and approximately 20% of patients with a Ct value of 26 in the acute phase then died. Likewise, those patients with a Ct value of 20 or 21 had a 50:50 chance of survival [1].

Using data on host transcripts and their abundance in combination with viral load information, sequencing approaches could be used to evaluate the efficacy of a medical countermeasure in a single compassionate use case [5]. However, the roots of this analysis hinged on the availability of facilities to process RNA from blood samples and access to high-resolution sequencing and performance computing. Therefore, identifying specific mRNA biomarkers, whose abundance can be measured using RT-qPCR, would be a useful tool in the evaluation of medical countermeasures using baseline survival data. This can be used in isolation or in combination with viral load information (which is measured during diagnostic confirmation).

This analysis includes a panel of 4 transcripts whose abundance in the blood in the acute phase of infection had been correlated with outcome [6, 16, 17]. The abundances of *VCAM1*, *TUBG1*, *MS4A4A*, and *ISG15* were taken forward for analysis with viral load information to predict clinical outcome. When used in combination with viral load data, the inclusion of host markers improved sensitivity and specificity for predicting either a fatal or survival outcome to more than 90%. Interestingly, within the gene set, *MS4A4A* was identified as a strong predictor of EVD fatality because the transcript abundance was greater in hospitalized fatal cases than hospitalized survivors and had a high accuracy power for classifying fatal cases without EBOV Ct values. Although the analysis of the predictive value of these host markers in an independent cohort of clinically ambiguous mid-range cases was not possible, a secondary analysis of a stratified subset with mid-range Ct values corroborated the strong prognostic value of *MS4A4A* as a standalone predictor and together with viral load. One potential explanation is the expression of *MS4A4A* in M2 polarized macrophages. This cell type has been associated with severe immunopathology in EVD and fatality [22]. Immune modulation is being explored as a viable way of enhancing survival in patients with EVD [23–25].

Patient data from the West Africa EBOV outbreak continue to provide a useful baseline for evaluating EVD. The identification of host biomarkers that can be measured by simple assays (RT-qPCR), in conjunction with viral load, to predict what the outcome would have been will help in the future evaluation of medical countermeasures and management of patients and care.

## Supplementary Material

jiag308_Supplementary_Data

## Supplementary Data

Supplementary materials are available at *The Journal of Infectious Diseases* online (http://jid.oxfordjournals.org/). Supplementary materials consist of data provided by the author that are published to benefit the reader. The posted materials are not copyedited. The contents of all supplementary data are the sole responsibility of the authors. Questions or messages regarding errors should be addressed to the author.
