# Supplementary material for: Identification of Host Gene Transcripts by Machine Learning and Their Application to Predict Outcome in Ebola Virus Disease
Source: J Infect Dis. 2026 Jun 17;234(4):e848–58. doi: 10.1093/infdis/jiag308 (PMC13668333; doi:10.1093/infdis/jiag308)
Supplement: Supplementary file 1 [file jiag308_supplementary_data.docx]

**VCAM1**

**TUBG1**

**MS4A4A**

**ISG15**

Supplementary Figure 1. Linearity and PCR efficiency of the optimized assays. Left panels show the amplification chart of the five cRNA standards for each gene, with dilutions ranging from 10^3^ to 10^7^ or 10^4^ to 10^8^ copies/µl and tested in triplicate. Right panels show the standard curves created with these dilutions. The mean Cq values are plotted against the log of concentration of the target gene copies/reaction. The PCR efficiency for each target was calculated using the slope of the calibration curve with the formula E = 10^−1/slope^−1. E = PCR efficiency, R^2^ = correlation coefficient.

**VCAM1	TUBG1**

**MS4A4A	ISG15**

Supplementary Figure 2. Melting curve analysis of the optimized RT-qPCR assays. The figures show the melting peaks of the RT-qPCR products obtained from the cRNA dilutions used to construct the standard curve. A single peak is visible for all primer pairs and genes tested.

**Supplementary Figure 3.** Distribution of the transcript abundance of the candidate predictive genes in clinical samples and corresponding EBOV Ct values.

**Supplementary Figure 4.** Evaluation of different machine learning algorithms in the whole dataset of patients. Accuracy is the percentage of correctly classified patients out of the whole dataset. Kappa is based on the accuracy but corrected for a chance agreement. Kappa values of zero or less indicates a bad performance of the classifier and values near to 1 indicates a good classifier. SVM = Support Vector Machine, KNN = k- Nearest Neighbor model, RF = Random Forest, BagC = Bagged CART, LRM = Logistic Regression Model, NB = Naive Bayes, C50 = C5.0, LDA = Linear Discriminant Analysis, GBM = Generalized Boosted Modeling, CART

**Supplementary Figure 5.** Confusion matrix showing SVM models built with a single gene transcript.

**Supplementary Figure 6.** Confusion matrix showing SVM models built with only three gene transcripts.

**Supplementary Figure 7.** Confusion matrix showing other SVM models with high predictive performance that include EBOV Ct values.

**Supplementary Figure 8.** Confusion matrix showing the performance of SVM models built with VCAM1, TUBG1 and ISG15 in the new testing set.

Supplementary Table 1. Performance evaluation of the optimized RT-qPCR assays for the four host potential predictors.

| Gene | Length of cRNA  (b) | qRT-PCR product size  (bp) | Slope | r^2^ value | PCR Efficiency (E) |
| --- | --- | --- | --- | --- | --- |
| VCAM1 | 1049 | 61 | -3.366 | 0.999 | 98.2% |
| TUBG1 | 1049 | 87 | -3.421 | 0.999 | 96.0% |
| MS4A4A | 1090 | 80 | -3.586 | 0.999 | 90.1% |
| ISG15 | 727 | 80 | -3.428 | 0.998 | 95.8% |

**Supplementary Table 2.** Correlation analysis of different variables in patient outcome showing the variables from the EMLab samples.

| Variable | Correlation coefficient (r) | P value | Bonferroni corrected P value |
| --- | --- | --- | --- |
| EBOV Ct | 0.5018238 | 1.82E-06 | 1.09E-05 |
| Age | -0.1190003 | 0.29 | 1.00 |
| VCAM1 | -0.4432559 | 3.41E-05 | 2.05E-04 |
| TUBG1 | 0.3269829 | 2.89E-03 | 0.02 |
| MS4A4A | -0.5119108 | 1.04E-06 | 6.22E-06 |
| ISG15 | -0.0697079 | 0.54 | 1.00 |

**Supplementary Table 3.** Correlation analysis of Favipiravir treatment in patient outcome showing the variables from the EMLab samples.

| Variable | Correlation coefficient (r) | P value | Bonferroni corrected P value |
| --- | --- | --- | --- |
| EBOV Ct | 0.555613 | 4.92E-07 | 2.95E-06 |
| Age | -0.16453 | 0.17 | 1.00 |
| VCAM1 | -0.54925 | 7.05E-07 | 4.23E-06 |
| TUBG1 | 0.04721 | 0.70 | 1.00 |
| MS4A4A | -0.59839 | 3.56E-08 | 2.14E-07 |
| ISG15 | 0.279988 | 0.02 | 0.11 |
